# Anti-inflammatory and antibacterial effects of human cathelicidin active fragment KR-12 in the mouse models of colitis: a novel potential therapy of inflammatory bowel diseases

**DOI:** 10.1007/s43440-020-00190-3

**Published:** 2020-11-21

**Authors:** Natalia Fabisiak, Adam Fabisiak, Anna Chmielowiec-Korzeniowska, Leszek Tymczyna, Wojciech Kamysz, Radzisław Kordek, Marta Bauer, Elżbieta Kamysz, Jakub Fichna

**Affiliations:** 1 Department of Biochemistry, Faculty of Medicine, Medical University of Lodz, Mazowiecka 6/8, 92-215 Lodz, Poland; 2 Department of Gastroenterology, Faculty of Medicine, Medical University of Lodz, Lodz, Poland; 3 Department of Digestive Tract Diseases, Faculty of Medicine, Medical University of Lodz, Lodz, Poland; 4 Department of Animal Hygiene and Environment, University of Agriculture in Lublin, Lublin, Poland; 5 Department of Inorganic Chemistry, Faculty of Pharmacy, Medical University of Gdansk, Gdansk, Poland; 6 Department of Pathology, Faculty of Medicine, Medical University of Lodz, Lodz, Poland; 7 Department of Molecular Biotechnology, Faculty of Chemistry, University of Gdansk, Gdansk, Poland

**Keywords:** KR-12, Inflammatory bowel disease, Crohn’s disease, Ulcerative colitis, Cathelicidins

## Abstract

**Introduction:**

Inflammatory bowel diseases (IBD) are a group of chronic gastrointestinal tract disorders with complex etiology, with intestinal dysbiosis as the most prominent factor. In this study, we assessed the anti-inflammatory and antibacterial actions of the human cathelicidin LL-37 and its shortest active fragment, KR-12 in the mouse models of colitis.

**Materials and methods:**

Mouse models of colitis induced by 2,4,6-trinitrobenzenesulfonic acid (TNBS) and dextran sulfate sodium (DSS) were used in the study. The extent of inflammation was evaluated based on the macro- and microscopic scores, quantification of myeloperoxidase (MPO) activity and microbiological analysis of stool samples.

**Results:**

A preliminary study with LL-37 and KR-12 (1 mg/kg, ip, twice daily) showed a decrease in macroscopic and ulcer scores in the acute TNBS-induced model of colitis. We observed that KR-12 (5 mg/kg, ip, twice daily) reduced microscopic and ulcer scores in the semi-chronic and chronic TNBS-induced models of colitis compared with inflamed mice. Furthermore, qualitative and quantitative changes in colonic microbiota were observed: KR-12 (5 mg/kg, ip, twice daily) decreased the overall number of bacteria, *Escherichia coli* and *coli* group bacteria. In the semi-chronic DSS-induced model, KR-12 attenuated intestinal inflammation as demonstrated by a reduction in macroscopic score and colon damage score and MPO activity.

**Conclusions:**

We demonstrated that KR-12 alleviates inflammation in four different mouse models of colitis what suggests KR-12 and cathelicidins as a whole are worth being considered as a potential therapeutic option in the treatment of IBD.

## Introduction

Inflammatory bowel diseases (IBD), which consist mainly of Crohn’s disease (CD) and ulcerative colitis (UC) is a group of chronic gastrointestinal (GI) tract disorders with multifactorial etiology. Genetic, immunological, and environmental factors are related to the pathogenesis of IBD [1]. The named diseases are characterized by a chronic course with periods of exacerbation and remission. Multiple treatment choices are available for patients with IBD what depends on disease activity and its course. In line, analogs of 5-aminosalicylic acid administered orally or rectally are basic drugs; glucocorticoids, immunomodulatory drugs, or biological therapies are used when remission has not been achieved. In treated patients, cumbersome side effects appear, such as hypokalemia, hypertension, diabetes mellitus (glucocorticoids), neurological disorders, and increased susceptibility to infections (biological therapy) [2]. In portion of patients, the therapy is not effective and a surgical intervention is necessary which sometimes may be debilitating. Therefore novel treatment options are still sought to treat patients more efficiently.

Human cathelicidin antimicrobial peptide (AMP), LL-37 (also known as h-CAP18) is the only cathelicidin found in humans [3]. LL-37 is synthesized in neutrophils, keratinocytes, epithelial cells of respiratory tract and bowels, and excreted onto human body surfaces remaining in contact with the external environment. Cathelicidins exhibit beneficial effect on the structure and function of the epithelium [4, 5]. The anti-inflammatory activity of cathelicidins was proven by Koon et al. in a rodent model of colitis [6]. Concurrently, cathelicidin-BF derived from the snake venoms, was shown to inhibit the expression of pro-inflammatory cytokines, such as TNF-α, IL-6 and IL-8, and to increase the expression of the anti-inflammatory cytokine IL-10 in the human intestine [7].

In recent years, the studies concentrated on identification of a shorter fragment of LL-37, which could possess antibacterial activity [8]. Currently, a peptide named KR-12–corresponding to residues 18–29 of LL-37–is known to be the shortest analog of human cathelicidin exhibiting antimicrobial activity [9]. Noteworthy, KR-12 displays similar antimicrobial activity against Gram-negative and Gram-positive bacteria as LL-37 [10].

Even though the intestinal dysbiosis plays a significant role in the development of IBD, current strategies to modulate the gut microbiome failed to induce any significant results in IBD treatment [11]. One of plausible options is by navigating the composition of the microbiota by naturally occurring peptides. In this study, we characterized the anti-inflammatory and antibacterial effects of KR-12 in the mouse models of colitis. The results of our study showed that KR-12 may be considered as a potential novel target in the treatment of colitis.

## Materials and methods

### Animals

Male BALB/c mice were obtained from the Animal House at the Nofer Institute of Occupational Medicine, Lodz, Poland. Mice weighing 22–26 g (6–8 weeks of age) were used in the experiments. All animals were housed in sawdust-lined plastic cages at a constant temperature (22 °C) and maintained under a 12 h light/dark cycle (lights on 6:00 a.m.). Access to chow pellets (Agrapol SJ., Motycz, Poland) and tap water ad libitum was assured. All animal care and experimental protocols were complied with the European Communities Council Directive (2010/63/EU) and Polish legislation acts concerning animal experimentation. The experimental protocol will be approved by the Local Ethics Committee at the Medical University of Lodz (36/ŁB98/2018). Efforts were made to minimize animal suffering and to reduce the number of animals used. Groups of 8–10 animals were used in all experiments.

### Drugs

In our experiment, we used two peptides LL-37 (LLGDFFRKSKEKIGKEFKRIVQRIKDFLRNLV-PRTES) and KR-12 (KRIVQRIKDFLR) which were synthesized manually by solid-phase method using 9-fluorenylmethoxycarbonyl (Fmoc) chemistry on a Wang resin (loading 0.50—mmol/g, 100–200 mesh, Merck, Germany) according to procedures described in our previous works [12, 13]. The purity of peptides after purification was at least 95%, as determined by analytical reversed-phase high-performance liquid chromatography (RP HPLC). Their identity was confirmed by the matrix-assisted laser desortion/ionization time-of-flight (MALDI TOF) mass spectrometry. All drugs were dissolved in 5% dimethyl sulfoxide (DMSO) in saline, which was used as vehicle.

### Induction of colitis

Colonic inflammation was induced by four various models as described earlier [14]. Briefly, mice were weighed and anesthesized with 1% isoflurane (Baxter Healthcare Corp., IL, USA). 2,4,6-trinitrobenzenesulfonic acid (TNBS, 0.1 mL of 30% ethanol in saline; (1) acute and (2) semi-chronic model: 4% TNBS; (3) chronic relapsing model: first dose of TNBS on the induction of colitis—150 mg/kg, second dose on day 11th of the experiment—75 mg/kg) was administered through a catheter inserted 3 cm proximally from the anus. Then, mice were maintained in an inclined position for 1 min to ensure a thorough distribution of the solution in the colon. After TNBS infusion recovery was allowed with food and water supplied. p animals received vehicle alone (0.1 mL of 30% ethanol in saline). The last (4) model of colitis was induced by dextran sulfate sodium (DSS 3% wt/vol; molecular weight 40,000; MP Biomedicals, Aurora, OH, Lot No. 5237 K) which animals received from day 0 to 5th. Starting from day 6th, DSS was replaced with tap water. Control animal have been receiving a tap of water during the whole experiment. Animal body weight and clinical symptoms of colitis (diarrhea and bloody stool) in mice were evaluated daily.

### Pharmacological treatment

**Fig. 1:**
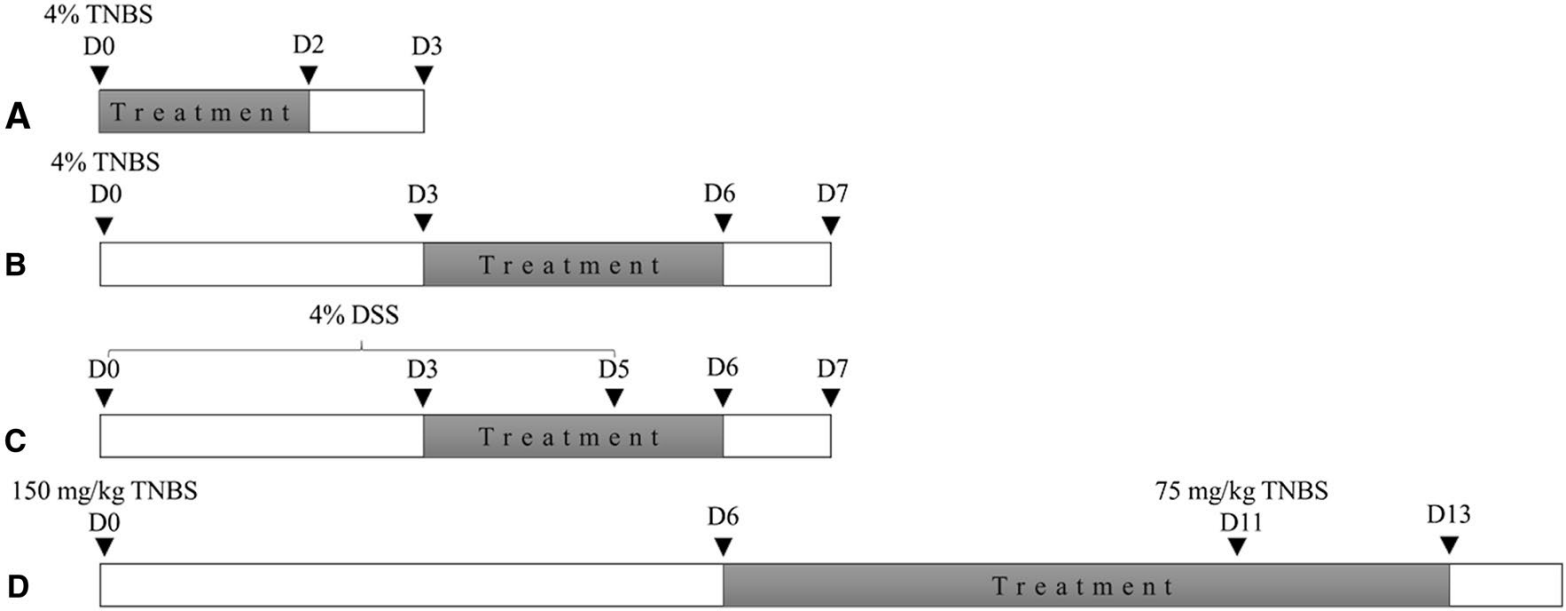
Scheme 1. Graphical presentation of four mouse models of experimental colitis used in the study: acute (**a**), semi-chronic (**b**) and chronic (**c**) TNBS-induced model of colitis, and (**d**) semi chronic DSS-induced model of colitis

In the acute TNBS-induced model of colitis, LL-37 or KR-12 was administered intraperitoneally (ip), twice daily (BID) at the dose of 1 mg/kg from day 0 to 2nd of the experiment. In the semi-chronic TNBS-induced model of colitis animals were treated with KR-12 (5 mg/kg, ip, BID) from day 3rd to 6th of the experiment. In the chronic TNBS-induced model, KR-12 was administered at the dose of 5 mg/kg, ip, BID from day 6th to 13th of the experiment. In the DSS-model of colitis, mice received KR-12 (5 mg/kg, ip, BID) from day 3rd to 6th of the experiment. Control animals received vehicle alone (100 μL, ip) in all experiments. Schemes of models are depicted in Fig. 1.

### Assessment of colonic damage

#### TNBS-induced colitis

On day 3rd in acute model, 7th in semi-chronic model and 14th in chronic model the animals were sacrificed by a rapid cervical dislocation and the evaluation of colonic damage was performed. After euthanasia, the total macroscopic score was assessed. The colon was rapidly removed, opened longitudinally, and rinsed with phosphate buffered saline (PBS) to remove the feces. Then immediate examination was performed as described earlier [14]. Macroscopic colonic damage was determined by an established semiquantitative scoring system by adding individual scores for ulcer, shortening of the colon, colonic wall thickness, and presence of hemorrhage, fecal blood, and diarrhea, as described before [14]. For scoring ulcer and colonic shortening the following scale was used: ulcer: 0.5 points for each 0.5 cm; shortening of the colon: 1 point for > 15%, 2 points for > 25%. The colonic wall thickness was measured in millimeters. A thickness of n mm corresponded to n scoring points. The presence of hemorrhage, fecal blood, or diarrhea increased the score by 1 point for each additional feature.

#### DSS-induced colitis

Disease parameters were evaluated on day 7 following addition of DSS to the drinking water. After euthanasia, the entire colon was isolated and weighed with fecal content. Then, colon was opened longitudinally and cleaned from the fecal material. A total macroscopic damage score was calculated for each animal based on the (1) stool consistency (where 0 means normal well-shaped fecal pellets and 3 means diarrhea), (2) colon epithelial damage considered as a number of ulcers (0–3), (3) colon length and weight scores expressed as a percentage loss of each parameter in relation to the control group (0 points, ≤ 5% weight/length loss; 1 point, 5–14% weight/length loss; 2 points, 15–24% weight/length loss; 3 points, 25–35% weight/length loss; and 4 points, ≥ 35% weight/length loss). Total score = 0 means no inflammation. The presence (score = 1) or absence (score = 0) of fecal blood was also recorded.

Samples were collected and kept in − 80 °C for further assessment.

### Determination of myeloperoxidase activity (MPO)

To assess granulocyte infiltration and to quantify the MPO activity we used the method described earlier [14]. Specimen of large colon with a minimum weight of 20 mg was provided and shortly, homogenized in hexadecyltrimethylammonium bromide (HTAB) buffer (0.5% HTAB in 50 mM potassium phosphate buffer, pH 6.0; 50 mg tissue/mL) immediately after isolation. Then, homogenate was centrifuged (15 min, 13,200 rpm, 4 °C) and 7 μL of supernatant was added on a 96-well plate followed by 200 μL of 50-mM potassium phosphate buffer (pH 6.0), containing 0.167 mg/mL of O-dianisidine hydrochloride and 0.05 μL of 1% hydrogen peroxide. Absorbance was measured at 450 nm (iMARK Microplate Reader, Biorad, United Kingdom) after 0, 30, and 60 s. All measurements were performed in triplicate. MPO was expressed in milliunits per gram of wet tissue, 1 unit being the quantity of enzyme able to convert 1 µmol of hydrogen peroxide to water in 1 min at room temperature. Units of MPO activity per 1 min were calculated from a standard curve using purified peroxidase enzyme.

### Histology

After the macroscopic scoring, the fragments of the distal colon (approximately 0.5 cm in length) were stapled flat, mucosal side up, onto cardboards and fixed in 10% neutral-buffered formalin for 24 h at 4 °C. Samples were dehydrated, embedded in paraffin, sectioned at 5 μm, mounted onto slides and stained with hematoxylin and eosin. Subsequently, sections were examined using microscope (Motic AE31 microscope, Ted Pella, Sweden). A digital imaging system consisting of a digital camera (Moticam 2300, Ted Pella, Sweden) and image analysis software (Motic Images Plus 2.0, Germany) were used to take photographs. A microscopic total damage score was assessed by a blinded fashion using the scoring system based on the presence (score = 1) or absence (score = 0) of goblet cell depletion, the presence (score = 1) or absence (score = 0) of crypt abscesses, the destruction of mucosal architecture (normal = 1, moderate = 2, extensive = 3), the extent of muscle thickening (normal = 1, moderate = 2, extensive = 3), and the presence and degree of immune cell infiltration (normal = 1, moderate = 2, transmural = 3).

### Microbiological assessment

The microbiological assessment was performed according to Siczek et al. [15]. Firstly, 5 mL of Ringer’s solution was added into the sterile falcons with mouse intestinal contents. Next, the material was centrifuged for 5 min and left for 15 min for sedimentation. Series of tenfold dilution were prepared in saline solution. From each series, 0.1 mL of the mixture was taken and cultures were performed in duplicates using superficial method in appropriate agar medium: enriched media (BTL Polska Sp. z o.o.) for count of overall bacteria, Endo les media (BTL Polska Sp. z o.o.) for the count of overall bacteria of coli strains, mFC media (BTL Polska Sp. z o.o.) for count of *Escherichia coli*, Iron sulfide agar media (Biomerieux Polska Sp. z o.o.) for count of bacteria of *Clostridium* strains, MRS media (BTL Polska Sp. z o.o.) for count of overall bacteria of *Lactobacillus* strains and Sabourada media (BTL Polska Sp. z o.o.) for count of overall fungi. Respective conditions were provided in each colony: to count the overall bacteria, bacteria of *coli* and *Clostridium* strains incubation was performed for 24–48 h in 37 oC; to count the overall bacteria of *Lactobacillus* strains incubation was performed for 3–5 days in 44 °C; to count the overall fungi the incubation was performed for 7 days in 25 °C. The cultures of *Clostridium* and *Lactobacillus* strains were incubated in controlled atmosphere using Genbag sachets (Biomerieux Polska Sp. z o.o.). After the specific time of incubation, the colonies were counted using automated method according to PN-ISO 4832 standards. Finally, the number of each morphological types was expressed as colonies forming units in 1 g of the mouse fecal content [cfu/g].

### Data analysis

Statistical analysis was performed using PRISM 8.0.1 (Graph-Pad Software Inc., La Jolla, CA, USA). Assumption of normal distribution of differences was verified with the use of Shapiro–Wilk test. ANOVA followed by Newman–Keuls test as a post hoc test was used for analysis of multiple treatment means when the normality test was passed. When the normality test was violated, the significance of differences was tested with the multiple comparison Kruskal–Wallis test to compare three or more independent groups. The data are expressed as means ± SEM or median with interquartile range. *p* values ≤ 0.05 were considered statistically significant.

## Results

### Acute TNBS-induced model of colitis

**Fig. 2:**
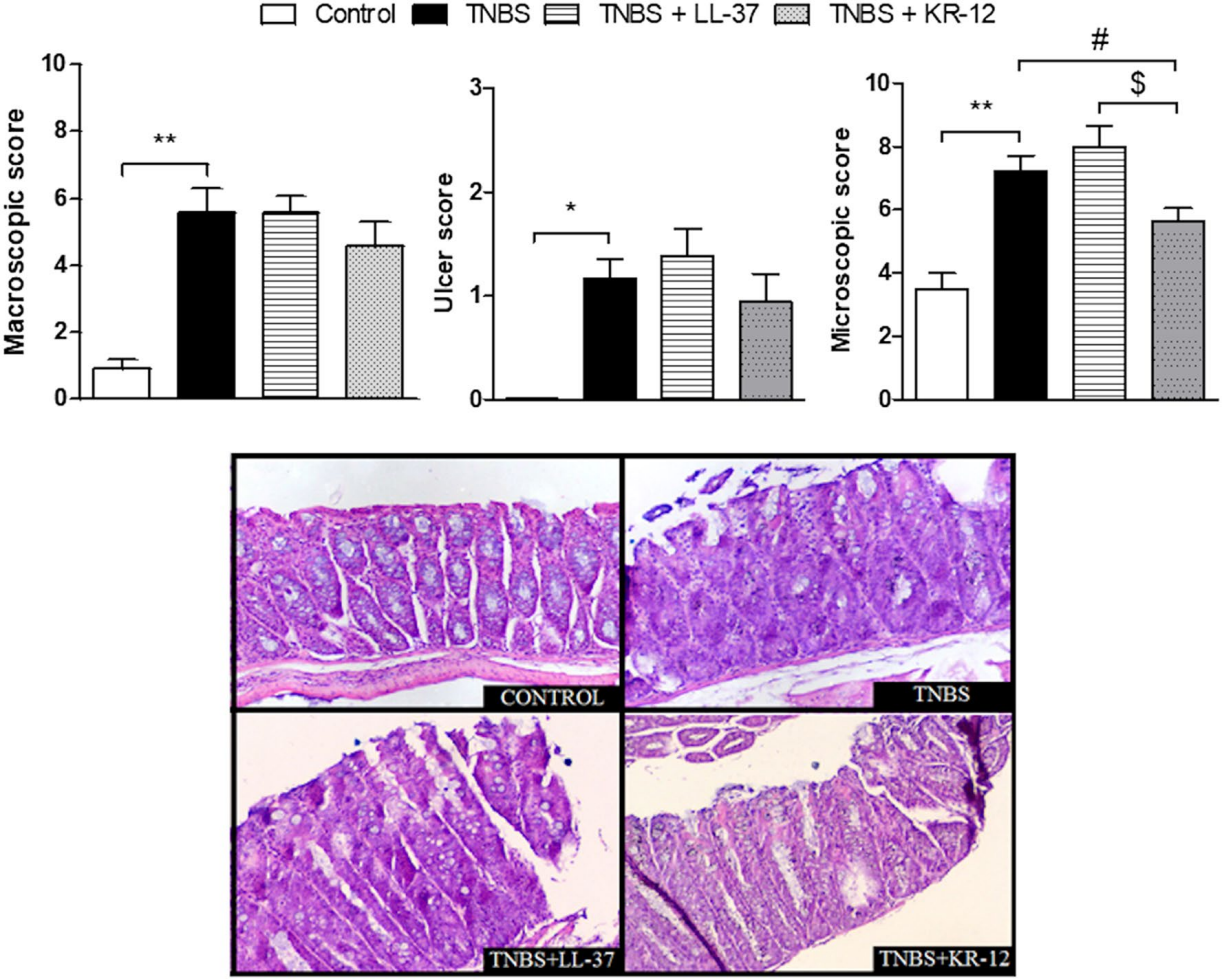
The effect of LL-37 and KR-12 (1 mg/kg, 100 µL/animal, ip, twice daily on days 0–2 from induction of colitis) on macroscopic, microscopic and ulcer scores in the acute model of colitis induced by TNBS. *p < 0.05, **p < 0.01 as compared to control mice; ^#^*p* < 0.05 as compared to TNBS-treated animals; ^$^*p* < 0.05 as compared to mice treated with LL-37. Data represent mean ± SEM of 8–10 mice per group. All data were distributed normally, one-way ANOVA test with Newman–Keuls as a post hoc test was used

We decided to run a preliminary experiment with both: LL-37 and KR-12 to compare their anti-inflammatory activity. Unlike LL-37, KR-12 (both 1 mg/kg, ip, BID) reduced macroscopic score compared to mice treated with TNBS (5.56 ± 0.49 and 4.55 ± 0.76 vs. 5.58 ± 0.71, respectively (*F*_3,25_ = 5.33, df = 28)). LL-37 increased, while KR-12 reduced the ulcer score compared to TNBS-treated mice [1.38 ± 0.26 and 0.93 ± 0.27 vs. 1.16 ± 0.18 (*F*_3,25_ = 3.28, df = 28)]. Accordingly, microscopic assessment showed a statistically significant decrease of histological damage after treatment of KR-12 compared to LL-37 and mice treated with TNBS [5.64 ± 0.40 vs. 8.00 ± 0.65 vs. 7.22 ± 0.48, *p* = 0.02 (*F*_3,23_ = 8.52, df = 26)] (Fig. 2).

### Semi-chronic and chronic TNBS-induced models of colitis

Based on preliminary results which evidenced potent anti-inflammatory action of KR-12 over LL-37, we decided to perform detailed studies solely with the shorter peptide. In the semi-chronic model, macroscopic assessment showed a reduction of intestinal damage after treatment with KR-12 (5 mg/kg, ip, BID) compared to TNBS-treated mice (1.32 ± 0.17 vs. 1.98 ± 0.23, (*F*_2,17_ = 6.01). Also, the peptide significantly decreased the ulcer score compared to mice treated with TNBS [0.50 (0.00–0.50) vs. 1.00 (1.00–1.00), *p* = 0.04 (H = 16.15, N_1_ = 6, N_2_ = 8, N_3_ = 6)].

**Fig. 3:**
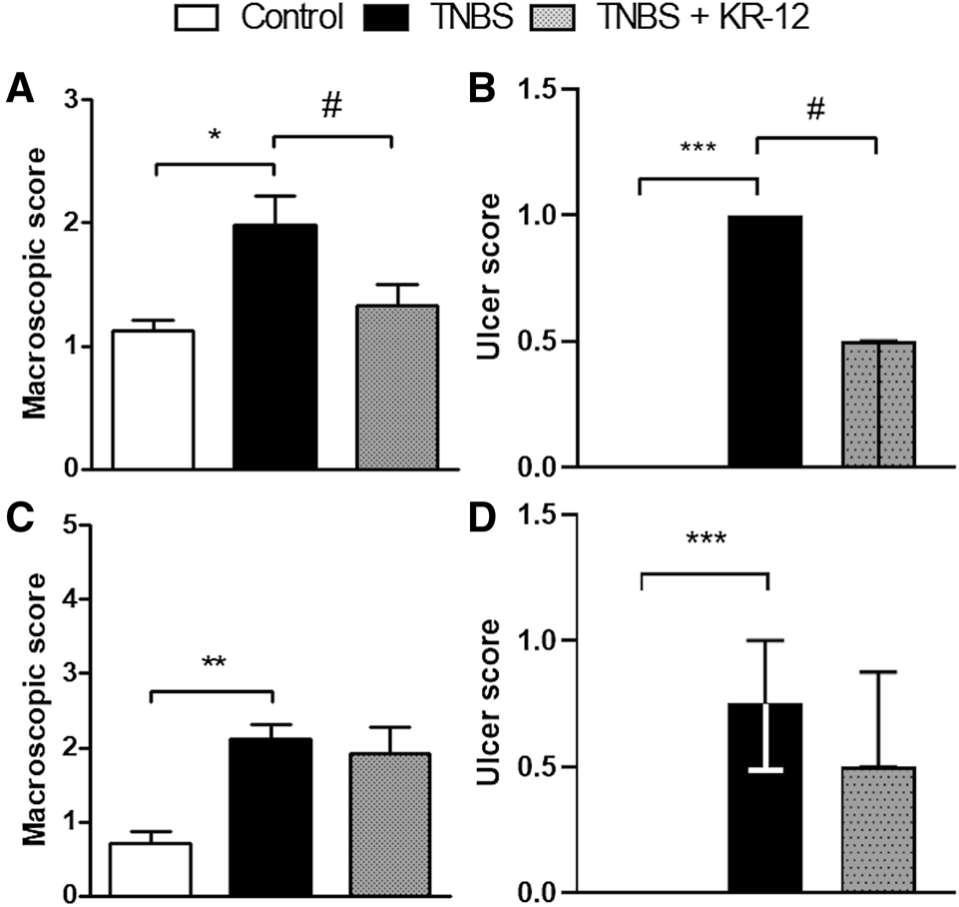
The effect of KR-12 on macroscopic and ulcer score in the semi-chronic (**a**–**b**) and chronic (**c**–**d**) models of colitis induced by TNBS. *p < 0.05, **p < 0.01, ***p < 0.001 as compared to control mice, ^#^*p* < 0.05, ^###^*p* < 0.001 as compared to TNBS-treated animals. In case of **a** and **c** statistics the data was distributed normally, one-way ANOVA with Newman-Keuls as a post hoc test was used. Data represent mean ± SEM of 8–10 mice per group. In case of **b** and **d** statistics, the data were not distributed normally and thus the Kruskal–Wallis test was used. Data represent median with interquartile range of 8–10 mice per group

Accordingly, in the chronic model of TNBS-induced colitis we observed a reduction in macroscopic score [1.92 ± 0.35 vs. 2.12 ± 0.18 (*F*_2,27_ = 5.40)] and ulcer score [0.50 (0.50–0.88) vs. 0.75 (0.50–1.00) (H = 14.25, N_1_ = 6, N_2_ = 12, N_3_ = 12)] after the treatment with KR-12 (5 mg/kg, ip, BID) compared with inflamed mice (Fig. 3).

### KR-12 influenced the microbiota of the mouse colon in the semi-chronic TNBS-induced model of colitis

In colonies cultured from TNBS-treated mouse stool samples we observed an increase in overall bacteria, *E. coli* and *coli* group compared to control mice (6.50 (2.10–11.10) cfu/g vs. 0.15 (0.11–4.73) cfu/g, 1.21 (0.00–3.16) cfu/g vs. 0.00 (0.00–0.12) cfu/g and 0.40 (0.00–9.31) cfu/g vs. 0.00 (0.00–0.29) cfu/g, respectively). In contrast, treatment with KR-12 (5 mg/kg, 100 µL/animal, ip, twice daily on days 3–6 from induction of colitis) decreased these counts (1.77 (0.83–3.02) for total count of bacteria (H = 6.25, N_1_ = 6, N_2_ = 8, N_3_ = 6), 0.00 (0.00–1.81) for *E. coli* (H = 4.42, N_1_ = 6, N_2_ = 8, N_3_ = 6) and 0.00 (0.00–5.10) for *coli* group (H = 3.64, N_1_ = 6, N_2_ = 8, N_3_ = 6). Fungi count was lowered in TNBS group and mice treated with KR-12 than in control group (0.00 (0.00–0.00) vs. 0.00 (0.00–0.09) cfu/g vs. 0.00 (0.00–0.37) cfu/g, respectively (H = 0.15, N_1_ = 6, N_2_ = 8, N_3_ = 6). No growth in cultures of *Lactobacillus* and *Clostridium* strains of bacteria was noted in any group. The effect of KR-12 on the colon microflora in the TNBS-induced semi-chronic colitis model is presented in Table 1.

### Semi-chronic DSS-induced colitis

**Fig. 4:**
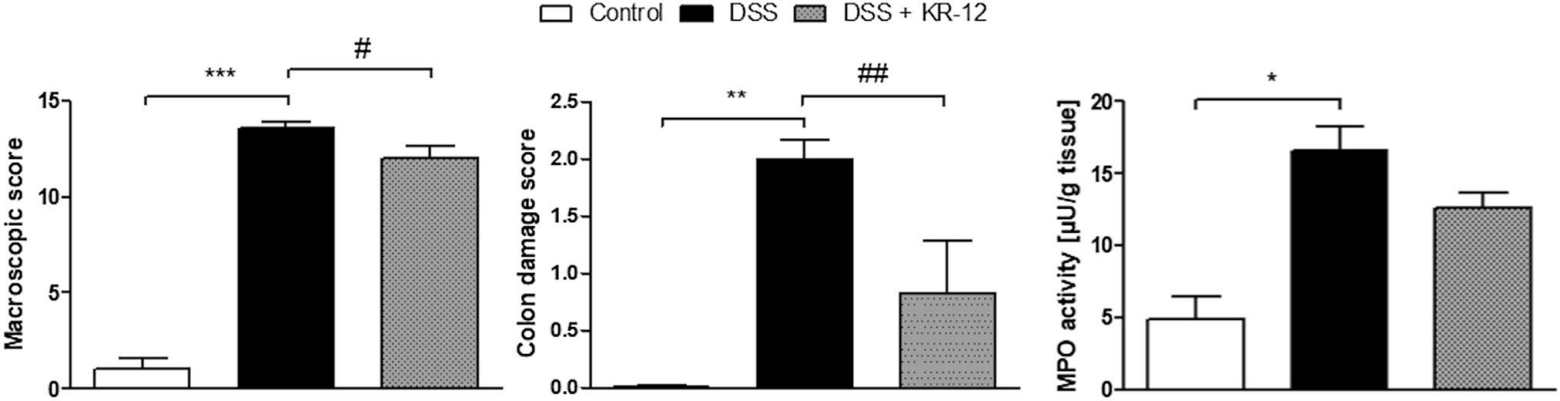
The effect of KR-12 on macroscopic, colon damage score and MPO activity in the semi-chronic model of colitis induced by DSS. *p < 0.05, **p < 0.01, ***p < 0.001 as compared to control mice, ^#^*p* < 0.05, ^##^*p* < 0.01 as compared to TNBS-treated animals. Data represent mean ± SEM of 8–10 mice per group. All data was distributed normally, one-way ANOVA test with Newman–Keuls as a post hoc test was used

Next, we wanted to explore the anti-inflammatory effect of KR-12 in the semi-chronic model of DSS-induced colitis. The peptide reduced macroscopic score (12.0 ± 0.671 vs. 13.6 ± 0.296, *F*_2,16_ = 127.70) and colon damage score (0.83 ± 0.46 vs. 2.0 ± 0.167, *F*_2,16_ = 10.21) compared to DSS-treated mice. KR-12 decreased MPO activity (12.62 ± 1.059 vs. 16.64 ± 1.69, *F*_2,14_ = 6.00) compared to mice treated with DSS (Fig. 4).

## Discussion

Strong evidences favor the significance of intestinal microbiota in the development of IBD. An overgrowth of some strains of bacteria (e.g., *Ruminococcus gnavas*, *Ruminococcus torques*) and a decrease in abundance of other (e.g., *Firmicutes*, *Bacteroidetes*) is observed in patients with IBD compared to healthy controls [11]. However, despite this evident role of dysbiosis in IBD, currently available approaches failed to induce any notable improvements in the treatment of patients with either CD or UC. Some studies hinted that the use of probiotics, especially of VSL#3 group, are effective in inducing remission in IBD [16], but we generally lack large randomized controlled trials and the data are not conclusive. Another strategy to reconstitute the composition of intestinal microflora laid in fecal microbiota transplantation (FMT). FMT was effectively adopted in guidelines of several societies as a treatment in refractory or hard-to-treat *Clostridioides difficile* infection [17]. Therefore, researchers encouraged by these results sought a potential in FMT as a treatment option of IBD. The studies for UC are sparse [18]; some found this approach effective in inducing remission [19], some failed to observe any response [20]. There are fewer studies regarding the utility of this method in CD. For example, Cui et al. [21] showed that the mid-gut transition of microbiota from healthy donors to patients with refractory CD is safe, feasible and effective; the meta-analysis of 11 trials of FMT in CD concluded that additional controlled studies are needed in this niche [22]. FMT method suffers strong limitations, as it is not yet standardized in case of route of administration or the transplant composition (Table 1).

**Table 1:** Effect of KR-12 (5 mg/kg, 100 µL/animal, ip, twice daily on days 3–6 from induction of colitis) on colonic microbiota in TNBS-induced semi-chronic model of colitis

Group	Total bacteria *n* × 10^7^	*Escherichia coli n* × 10^5^	Coli group bacteria	Fungi
Control	0.15 (0.11–4.73)	0.00 (0.00–0.12)	0.00 (0.00–0.29)	0.00 (0.00–0.37)
TNBS	6.50 (2.10–11.10)	1.21 (0.00–3.16)	0.40 (0.00–9.31)	0.00 (0.00–0.00)
TNBS + KR-12	1.77 (0.83–3.02)	0.00 (0.00–1.81)	0.00 (0.00–5.10)	0.00 (0.00–0.09)

Number of bacteria shown as number of colony forming units per gram of stool [cfu/g]. As the data were not distributed normally, Kruskal–Wallis test was used for statistics. Data represented in cfu/g of stool content as median with interquartile range of 6–8 mice per group

In our study, we tested yet another approach to restore physiological microbiota, i.e., by employing antimicrobial peptides. To date, only a few studies addressed the potency of LL-37 in IBD. Apart from its antimicrobial activity [23], the peptide was showed to possess pronounced pleiotropic functions including anti-inflammatory action (reviewed in [24]). With regard to IBD, Shauber et al. [25] found that the expression of LL-37 was increased in patients with UC, but not with CD. Contrary results were reported in a study conducted by Tran et al. [26], what brought to the conclusion that the circulating LL-37 level may be useful as a non-invasive predictor of mucosal damage when co-assessed with C-reactive protein in UC patients, a predictor of stricturing pattern of CD and an indicator of a better prognosis in IBD overall. Sparse data on LL-37 may emerge from the fact that the peptide is unstable in vivo due to the vulnerability to proteases limiting its half-life time [27]. Thus, novel analogs are under development. A recent article [28] showed the potential of LTA, an eight-component hybrid peptide including LL-37. LTA reduced the lipopolysaccharide (LPS)-mediated histological damage, decreased the level of TNF-α, IFN-γ, IL-6 and IL-1β and enhanced the intestinal permeability by increasing the expression of certain tight junction proteins in LPS-induced murine model of colitis.

KR-12 is the shortest known fragment of LL-37 with retained ability to eradicate bacteria [9] and preserved anti-inflammatory properties [29]. Although some studies addressed the potential utilization of LL-37 in the treatment of IBD, no studies on KR-12 exist. For this reason, in our study, we sought the anti-inflammatory action of KR-12 in chemically induced models of colitis.

First, we performed a screening study in the acute model of TNBS-induced colitis to compare the anti-inflammatory activities of LL-37 and its derivate, KR-12. We found that the latter was superior in alleviating inflammation in this model and thus it was chosen for further experiments. We confirmed the anti-inflammatory action of KR-12 in the semi-chronic and chronic models of colitis induced by TNBS and the semi-chronic model of colitis induced by DSS. Surprisingly, no action on MPO activity was found in TNBS-induced animal models, while in DSS-induced colitis KR-12 decreased this marker of neutrophil infiltration. This suggests that KR-12 mediates different activity depending on the model. Lastly, we confirmed the anti-microbial activity of KR-12 as showed by a decrease in total bacteria, bacteria of *E. coli* and *coli* group.

The results obtained in our study differ slightly from the outcomes reported by the group of Tai et al. Namely, they showed that mouse cathelicidin-related antimicrobial peptide (mCRAMP) given intrarectally at the dose of 2.5 and 5 mg/kg once daily for seven consecutive days after the induction of inflammation prevents dose-dependently the development of DSS-induced colitis, but has no influence on severity of intestinal inflammation when the treatment was initiated seven days after the induction of colitis [30]. Authors found several mechanisms responsible for the anti-inflammatory effect of mCRAMP including antimicrobial properties, the ability to enhance mucin gene expression and suppression of apoptosis caused by DSS. Furthermore, in another study using the DSS-induced mouse model of colitis [31], the group observed that a single rectal administration of a mCRAMP-expressing plasmid is as effective as repeated administration of the peptide for five consecutive days in mice deprived of the gene expressing cathelicidin. Taken together, these results suggest protective role of cathelicidins rather than their healing properties. In contrast, in our research, we succeeded in achieving the healing effect of KR-12 in used models of colitis. Several factors may have influenced this discrepancy between our study and the studies of Tai et al. First of all, we used the analog of human cathelicidin rather than the species-specific AMP, but evidence of this approach as legitimated already exists: for example Hosoda et al. [32] effectively suppressed the inflammatory response in murine model of sepsis by intravenous administration of LL-37 what strengthens our findings on possible use of KR-12 as a drug in future. Other possible reasons include different chemically induced models of colitis used or route of AMP administration.

In contrast to its parent peptide, the exact mechanisms underlying the anti-inflammatory activity of KR-12 was not yet assessed. LL-37 was found to induce plentiful of changes leading to attenuation of inflammation. The peptide was reported to interact with P2X7 receptor with known implication in ATP-mediated cell death and inflammation [33]. Human cathelicidin is also implicated in preserving the mucosal integrity intact by: (1) aiding the differentiation of epithelial cells [34, 35], (2) stimulating the mucus synthesis through mitogen-activated protein kinase activation and increase in expression of MUC gene [36].

Our study carries several strengths. Primarily, we managed to show that the shorter fragment of human cathelicidin is more effective as an anti-inflammatory drug than the parent peptide and proved our hypothesis in three different models of colitis. Moreover, KR-12 exhibited anti-inflammatory action via systemic administration what is crucial when searching for novel therapies. To date, LL-37 was the most effectively used when given topically. For example, a randomized controlled trial conducted by Grönberg et al. [37] revealed that the human cathelicidin administered on venous leg ulcer at the dose 0.5 or 1.6 mg/mL, twice weekly markedly enhanced the wound healing compared to placebo. Our results suggest that KR-12 with systemic administration is more stable and bioavailable than LL-37 which has a half-life time of about 1 h [38]. Moreover, as dysbiosis is crucial in the pathogenesis of IBD, we performed basic microbiological testing and found tested compound to effectively influence the intestinal microbiota of inflamed mouse colons. We hope our encouraging results will pave the way for the development of future treatment options for intestinal inflammation based on KR-12.

## Abbreviations

AMP: Antimicrobial peptide
BID: Twice daily
CD: Crohn’s disease
DSS: Dextran sulfate sodium
FMT: Fecal microbiota transplantation
GI: Gastrointestinal
ip: Intraperitoneally
IBD: Inflammatory bowel disease
LPS: Iipopolysaccharide
MPO: Myeloperoxidase
PBS: Phosphate-buffered saline
TNBS: 2,4,6-Trinitrobenzenesulfonic acid
UC: Ulcerative colitis
